# Foreign direct investment and domestic innovation: Roles of absorptive capacity, quality of regulations and property rights

**DOI:** 10.1371/journal.pone.0298913

**Published:** 2024-03-08

**Authors:** Atif Rao, Muhammad Ali, Jason M. Smith

**Affiliations:** 1 Department of Agricultural Economics, Kansas State University, Manhattan, KS, United States of America; 2 Chair of Micro Economics, Friedrich Schiller University, Jena, Germany; 3 Department of Economics and Finance, Jon M. Huntsman School of Business, Utah State University, Logan, UT, United States of America; Palestine Technical University - Kadoorie, STATE OF PALESTINE

## Abstract

Foreign Direct Investment is theoretically expected to facilitate the transfer of knowledge from the home country to the host country, however, the empirical evidence on the subject is mixed. Some studies have shown that, on one hand, as competition grows, the incentive to innovate reduces with the decrease in monopoly rents (Schumpeterian effect). On the other hand, market competition can also boost investments in R&D activities incentivized by incremental profits (Escape-Competition effect). Therefore, this study aims to explore which of these two effects dominates in the selected group of countries. This study also identifies the moderators of the relationship between FDI stock and domestic innovation. It examines the role of absorptive capacity, quality of regulations, and property rights protection in the innovative activities of the host countries. Generalized Method of Moments is used to estimate the parameters of the multivariate regression equation. The analysis is based on panel data consisting of 49 countries over 14 years. The results show that FDI has a negative relationship with domestic innovation, indicating the presence of the Schumpeterian effect. The extensions of the main models show that FDI positively affects domestic innovation in countries with higher absorptive capacity, the superior quality of regulation, and stronger protection of property rights. This study shows that the positive relationship between FDI and domestic innovation is conditional on the ability to absorb knowledge and quality of governance in the recipient countries.

## 1 Introduction

In a continuously evolving world today, innovation plays an important role in keeping pace with the evolution of firms and countries alike. The importance of innovation has been recognized since the times of Schumpeter and further developed by prominent authors such as [1]. The literature on innovation explains that innovation is regarded as the engine of the country’s economic growth [2]. Moreover, knowledge is the key determinant of innovation, and knowledge accumulation is not just an outcome of in-house research and development (R&D) and education-related efforts, but also includes knowledge produced outside the boundaries of an enterprise [3]. For instance, imports, multinational firms, the immigration of highly skilled workers, and international patent collaborations are considered important sources of international knowledge spillovers. Due to its tacitness, knowledge becomes geographically localized [4]; however, foreign direct investment has been shown to reduce this geographic localization of international knowledge diffusion [5–7]. Realizing the importance of innovation, our key policy question is “how to promote innovation?”. In particular, we focus on FDI as a source of knowledge spillovers and assess whether FDI inflows increase domestic innovation in the selected countries.

In literature, FDI by Multinational Enterprises is regarded as the most researched topic in global businesses [8]. Although FDI is theoretically expected to facilitate the transfer of knowledge from the home country to the host country, however, the empirical evidence on the subject is mixed. For instance, few studies show that FDI is positively associated with knowledge spillovers [9–14]. However, some studies also show that FDI has a negative relationship with domestic productivity [15–17], and have a non-linear relationship between market competition and innovation [18,19]. In addition to these competing conclusions, some studies have also shown contrasting effects of competition on innovation. On one hand, as competition grows, the incentive to innovate reduces with the decrease in monopoly rents (Schumpeterian effect). On the other hand, market competition can also boost investments in R&D activities incentivized by incremental profits (Escape-Competition effect) [20]. Due to uncertainty in FDI and innovation association, this study explores the relationship between FDI inflows and domestic innovation. Additionally, it also studies potential moderating variables including 1) Absorptive Capacity (AC), 2) Quality of Regulations (QoR) and 3) Protection of Property Rights (PoPR), that can strengthen the relationship between FDI and domestic innovation.

The first moderating variable, the level of absorptive capacity, can determine the extent to which the host country can benefit from FDI-related knowledge spillovers. The absorption of knowledge through technological spillovers and positive externalities through FDI requires local AC [21]. At the firm level, an employee’s level of education, for example, has been shown to have positive effects on innovation [22–24]. The second moderating variable is the Quality of regulations which has become one of the significant factors that explain differences in development across countries [25]. Recent empirical evidence explains that institutional differences affect the level of R&D spillovers. For instance, d’Agostino and Scarlato [26] show that inclusive institutions have a positive association with innovation. However, the host country differences in the institutional regulations have a negative effect on foreign subsidiary performance [27]. The third moderating variable, the Protection of property rights in the host country, also plays an important role in encouraging innovation in the country [28] but empirical evidence on PoPR and Innovation is also ambiguous. For instance, PoPR and R&D activities focused on innovation are positively correlated [29,30]. However, some studies also found a negative association between PoPR and innovation [31,32].

Due to the mixed nature of the relationship between FDI and innovation, this paper aims to analyze and address gaps in the existing literature. Particularly, we identify three preconditions that could fill these gaps and potentially influence the relationship between FDI and innovation: 1) absorptive capacity (proxied by human capital), 2) quality of regulations and 3) protection of property rights. Using panel data for 49 countries across 14 years, our findings suggest that FDI has a negative relationship with domestic innovation, similar to the findings of Brambilla et al. (2009) and Girma et al. (2006). However, in countries where AC, QoR, and PoPR are high, the relationship between FDI and domestic innovation is positive.

The rest of the paper is organized as follows. The next section describes the relationship between FDI and innovation followed by a description of moderating variables and formulation of hypotheses followed by the section on econometric models, data, and methodology. The final two sections discuss results and conclusions, respectively.

### 2 Theoretical background

A theoretical literature study on the economic literature’s determinants of innovation includes a range of theories and models that aim to elucidate the forces that propel and impede innovative endeavors. At the core of this discussion is Schumpeter’s theory of economic development, which maintains that innovation is the essential component of economic change and is primarily driven by entrepreneurial activity [33]. Schumpeter asserts that foreign direct investment (FDI) can help transfer technology and knowledge from investing (often developed) to recipient (often developing) countries. In line with Schumpeter’s emphasis on innovation as a crucial driver of economic growth and development, this transfer may promote local innovation. Furthermore, foreign direct investment (FDI) brings in new rivals to a market, which could spark a process of creative destruction. This can compel regional businesses to innovate to thrive, hence enhancing the relationship between innovation and economic growth. Foreign companies’ presence through FDI can encourage local entrepreneurship in two ways: either by raising rivalry, which pushes local businesses to develop or by having demonstration effects, which teach and adapt foreign technologies and techniques to local businesses.

Another significant contribution is the evolutionary theory of economic development, which was developed by Nelson and Winter [34] and highlights the impact of institutional factors and routine behaviors on innovation. The Schumpeterian idea of innovation is significantly expanded upon by Nelson and Winter’s evolutionary theory where they emphasized the idea that "routines" are the building blocks of organizational behavior—essentially, the company’s genetic makeup. These practices, which change throughout time, can involve everything from production methods to management strategies. Multinational corporations provide their distinct practices and skills to host nations through foreign direct investment (FDI), which may have an impact on local businesses’ daily operations and foster innovation. According to their methodology, market forces, variety (new concepts and technologies), selection (market pressures), and retention (successful inventions are maintained and expanded upon) all play significant roles in the evolutionary process of economic transformation. This evolutionary perspective can be used to comprehend how foreign direct investment (FDI) affects innovation in host nations, as foreign companies bring fresh variants and competitive pressures that stimulate domestic innovation. Nelson and Winter [34] places significant emphasis on learning and adaptation as catalysts for innovation. This viewpoint is essential for comprehending how FDI affects regional innovation ecosystems. Through FDI, workers and local businesses can adapt foreign technologies, acquire new skills, and increase their capacity for innovation. Furthermore, they draw attention to how institutional and environmental factors influence the processes of innovation. This indicates that in terms of FDI, the influence on domestic innovation depends not just on the foreign investment per se, but also on the institutional and regulatory framework locally, cultural factors, and pre-existing industrial structures.

Romer [35] expanded on Schumpeter’s theory with his endogenous growth theory. Romer notably emphasized the significance of knowledge and technology as important drivers of economic progress, building on Schumpeter’s theories about the role of creativity and innovation in economic development. His concept has important ramifications for comprehending how innovation and foreign direct investment (FDI) interact. Romer’s model views knowledge as a crucial input in the non-rival production function, meaning that the application of information by one individual does not exclude the use of it by others. This viewpoint emphasizes the significance of knowledge spillovers, a notion that is extremely pertinent to foreign direct investment. FDI frequently brings cutting-edge technologies and expertise to the host countries, which can benefit local businesses and boost the nation’s economy as a whole. Romer’s concept also emphasizes how important it is for governments to create a creatively stimulating atmosphere. Policies that guarantee political and economic stability, safeguard intellectual property rights, and build infrastructure, for example, can all attract foreign direct investment (FDI) and create an atmosphere that is conducive to innovation. Romer also underlined the importance of human capital (skills, education, etc.) in the innovation process and how FDI can impact the growth of human capital in host nations through jobs, training, and education, all of which can contribute to increased capacity for innovation.

In the empirical literature, Foreign direct investment is considered to be among the major channels of technology transfer [36]. Domestic firms improve productivity through knowledge spillovers from foreign direct investment [37,38]. Several studies have shown that FDI generates positive knowledge spillovers [1,12,39,40] and increases technology transfers [41–43]. FDI has also been found to have a positive relationship with innovation [11,44]. On the contrary, some studies also point towards the possible negative effects of FDI on innovation in the host country [17,45,46]. The reason for mixed findings could be the differences in the economic and institutional factors among countries. Therefore, we examine whether absorptive capacity, protection of property rights, and quality of regulations moderate the relationship between FDI inflows and domestic innovation. The following subsections explain the importance of these moderators in light of the existing studies.

### Role of absorptive capacity

Absorptive Capacity (human capital) is regarded as the theme of the WIPO [47] with the tagline ‘Human Factor in Innovation’. Moreover, this theme explores the role of the human factor including creators and individual innovators behind the innovation process. The appropriation of knowledge from technologically intensive imports or FDI requires a certain level of absorptive capacity [48,49]. AC determines the ability of the firms to identify, assimilate, and apply knowledge to produce novel products, processes, and services [50]. AC can be represented by human capital i.e. a country with higher human capital will have a better ability to absorb knowledge. Previous studies have shown that the education of employees and innovation are positively correlated [22,23,24]. Absorptive capacity is also a significant moderator of the relationship between FDI and innovation as most of the determining factors of this relationship fall under the domain of absorptive capacity [51,52]. Even for poverty alleviation through FDI, Arogundade et al., [53] show that the impact of FDI on poverty depends on the level of absorptive capacity in the host country. It is therefore important to understand whether, at the macro level, higher absorptive capacity helps countries to absorb knowledge flows from FDI. We hypothesize that higher AC positively moderates the relationship between FDI and Innovation.

### Role of quality of regulation

North [54] defines institutions as “rules of the game in a society”. The quality of institutional regulation has become an important factor in the development of countries and plays an essential role in attracting FDI and stimulating innovation in countries. According to Tebaldi and Elmslie [55], institutional reforms have a direct impact on enterprises to get incentives for R&D activities for knowledge creation. Moreover, Barro [25] stated that economic and political institutions have become significant factors to explain differences in development across countries. Sala-i-Martin [56] presented a pragmatic notion of institutions in terms of elements that permit societies in a way to work in their economy and modern capitalism. Inclusive institutions are important to attract FDI, however, many countries are facing difficulties in producing inclusive institutions. For instance, Acemoglu and Robinson [57] argued that technology advancement poses a threat to ruling parties and such parties thereby preventing the facilitation of MNC’s technological progress and innovation to preserve their political power. Also, Dias and McDermott [58] provide evidence that interest groups who are engaged in rent-protecting activities may lead to decreased innovation and growth through the structural barrier (rent-seeking) in economies. In their analysis of the FDI and growth nexus, Dada and Abanikanda [59] show that good institutional quality affects the magnitude of the impact of FDI on economic growth. Thus, the lack of inclusive institutions can be a significant factor in low innovation output.

In the existing literature, the role of institutions was more focused on ascertaining technological changes in innovation systems. More recent empirical evidence explains that institutional differences affect the level of R&D spillovers. For instance, d’Agostino and Scarlato [26] show that inclusive institutions have a positive association with innovation. However, the host country’s differences from the home country in terms of institutional regulations harm foreign subsidiary performance [27]. Due to uncertainties in the institutional regulations of the host country, the extra cost is associated with the home country’s subsidies to learn the new environment’s “rule of the game” [54]. Therefore, we assume that QoR moderates the relationship between FDI and innovation.

### Role of property right protection

From a policy perspective, knowledge spillovers are attractive for domestic production. However, multinational companies see knowledge spillovers as a threat to their competitive advantage, as domestic firms learn to imitate their products and services. PoPR in such a case protects innovators’ competitive knowledge, allowing innovators to personalize the benefits of their innovation for the specified period. PoPR provides a secure environment for FDI [60,61] shows that lack of PoPR is a significant barrier against local innovations. Lin et al, [30] also found that PoPR and corporate R&D activities are significantly and positively associated in China. Contrary to those in favor of PoPR, opponents of the concept argue that strong PoPR encourages monopolies, restricts the flow of knowledge, and wastes domestic resources by increasing the cost of imitation [62]. Innovators must recover their production costs to continue their production of products and ideas however cheap copying makes it difficult for the innovators to cover their production costs. In cases where very similar products can be introduced in the market with copyright protection, the social surplus is higher in case of no protection as compared to the copyright regime.

Similarly, PoPR is shown to be negatively associated with innovation [31,32]. The literature on the role of PoPR in knowledge flows is therefore inconclusive [63,64], making it important to understand how PoPR affects knowledge accumulation and innovation through FDI. We hypothesize that strong PoPR would restrict domestic firms from using the knowledge gained through knowledge spillovers.

**Hypothesis 1:** AC, QoR, and PoPR as important external moderators strengthen the association between FDI and innovation.

## 3 Econometric model and methodology

We begin with a simple Cobb-Douglas function for innovation where innovation out is a function of R&D capital stock (which functions like capital in a traditional production function) and human capital (which mimics labor input in a production function):

$$P_{i,t}=R\&D\;Capital\;{Stock}_{i,t}^{\alpha }*{HC}_{i,t}^{\beta }$$ (1)

Where the subscripts ‘i’ and ‘t’ denote the cross-sections (countries) and time-periods, respectively. The dependent variable ‘*P*_*it*_’ is the aggregated number of country patents by residents. Taking natural log on both sides we get:

$${lnP}_{i,t}=\alpha \;\ln\left({R\&D\;Capital\;Stock}_{i,t}\right)+\beta \;ln({HC}_{i,t})$$ (2)

Next, we add the variables of interest, FDI, PoPR, QoR and FC in Eq 2 and develop an autoregressive econometric model in Eq 3.


$${lnP}_{it}=\gamma_{0}+\gamma_{1}ln(P_{it-1})+\alpha \;ln(R\&D\;Stock_{it})+\beta \;ln(HC_{it})+\gamma_{2}\;ln(FDI_{it})+\gamma_{3}\;ln({PoPR}_{it})+\gamma_{4}\;{ln(QoR}_{it})+\gamma_{5}FC+\epsilon_{it}$$ (3)

All variables are used in their natural logarithmic forms for ease of interpretation and accounting for outliers. FDI represents the stock of Foreign Direct Investment in the host country, PoPR represents Protection of Property Rights, QoR represents Quality of Regulations, and FC is a dummy for the financial crisis which equals one for the year 2008 and zero otherwise. The econometric model presented in Eq 3 resembles the model of Chen [12] which finds a positive association of FDI with the number of domestic patents using Chinese provincial data. In the extended versions of Eq 3, the interactions between FDI & AC, FDI & QoR, and FDI & PoPR are included sequentially in separate models to test Hypotheses 1.

We suspect bidirectional causality in Model 3. Knowledge-seeking FDI takes place in places where innovation is high, therefore, patenting can reverse cause FDI. Similarly, successful innovation incentivizes further investment in R&D, enabling reverse causality. Moreover, in panel data, the autoregressive term is correlated with the fixed effects in the error term, which gives rise to dynamic panel (or Nickell) bias [65]. A fixed-effects model will provide inaccurate results in the presence of dynamic panel bias. The Generalized Method of Moments (GMM) estimation procedure addresses the problem of endogeneity as well as dynamic panel bias (see [66,67] for details). The system GMM estimates the system of equations using the lags of endogenous variables in the first stage to account for the endogeneity problem. For strictly exogenous variables, one-year and longer iv-style instruments are used, for weakly exogenous variables, one-year and longer gmm-style instruments are used, and for the endogenous variables, two-year and longer gmm-style instruments are used. Therefore, to reduce the problem of endogeneity in our analysis, we use the Generalized Method of Moments (GMM) to estimate the parameters of Eq 3. We expect that the protection of property rights and quality of regulations may not be caused by the number of patents. Therefore, these two determinants are considered strictly exogenous. All the other variables are likely to be reverse caused by patents, therefore, they are treated as endogenous variables.

## 4 Variable definitions and data sources

This section explains the variables and provides sources of data used in this study. The estimations are based on the data from 49 countries between 2001 and 2021. List of countries is provided in Table 1. Definitions of variables and sources of data are presented below.

**Table 1:** Countries included in the analysis.

S. No.	Country Name	S. No.	Country Name
1	Argentina	27	Mexico
2	Australia	28	Moldova
3	Belgium	29	Netherland
4	Brazil	30	New Zealand
5	Bulgaria	31	Norway
6	Canada	32	Pakistan
7	China	33	Paraguay
8	Colombia	34	Poland
9	Czech Republic	35	Portugal
10	Denmark	36	Romania
11	Estonia	37	Russia
12	Finland	38	Singapore
13	France	39	Slovakia Republic
14	Germany	40	Slovenia
15	Hungary	41	South Africa
16	Iceland	42	Spain
17	India	43	Sri lanka
18	Iran	44	Sweden
19	Ireland	45	Thailand
20	Israel	46	Turkey
21	Italy	47	Ukraine
22	Japan	48	United Kingdom
23	Korea	49	United States
24	Lithuania		
25	Malaysia		
26	Malta		

According to the World Bank definition, “patent (P) is a process that gives a technical solution and new techniques for the problems in hand, it provides monopoly rights to the patent holder for a specific time”; generally, it is 20 years. Following Jaffe [68] and Cheung and Lin [11], this study uses the “number of patent applications by residents” as a proxy for domestic innovation.

Knowledge embodied in knowledge-intensive investment has a long-term life span, therefore instead of using the flows of foreign direct investment (FDI), this study uses FDI stock. To account for the differences in the size of the economies, the FDI variable is taken as a percentage of GDP. The source of FDI stock data is UNCTAD statistics.

This study uses two proxies for absorptive capacity (AC); 1) human capital and 2) research and development capital stock. The human capital index of Penn World Tables V.9 is based on average years of schooling which only represents the general level of education in the country, but it does not necessarily show the ability to innovate. While on the other hand, R&D capital stock represents the stock of knowledge that is directly related to the ability to innovate or absorb external knowledge related to technological innovation. For this reason, two proxies are used to compare the effects of different types of absorptive capacities on innovation. R&D capital stock is calculated using R&D flow variable from World Development Indicators using perpetual inventory method assuming 5% depreciation rate.

Protection of property rights (PoPR) is regarded as a central element of economic freedom. Fraser institute has developed the data for PoPR and this measure is ranked from 1 to 10 where10 represents high rank (highly protected) and 1 is low rank (low protection). This PoPR proxy is taken from the second component of economic freedom named “Legal system and Property rights” and the subcategory name is “2C Protection of Property rights” (see Gwartney et al., [69] for details).

The Quality of Regulations (QoR) in the country is concerned as an important element of the economic freedom index. This QoR proxy is taken from the fifth component of economic freedom named “Regulations”. Also, this measure covers the three subcomponents including credit, labor, and business market regulations. QoR is also ranked from 1 to 10 where 10 is high rank (high quality of regulations) and 1 is low rank (poor quality of regulations) (see Gwartney et al., [69] for details).

Variable definitions, data sources, and expected signs are summarized in Table 2. The coefficient of Foreign Direct Investment can have either positive or negative signs depending on the underlying dynamics explained in the conceptual background above. Similarly, QoR and PoPR can also have either positive or negative signs. Finally, we expect absorptive capacity to positively affect domestic innovation.

**Table 2:** Definition and sources of variables.

Variable names	Symbols	Proxy	Source	Expected sign
Domestic innovation (patents)	P	Patent applications by residents	World Development Indicators (WDI), The World Bank.	
Foreign direct investment inflows	FDI	FDI inflows stock percentage of GDP	UNCTAD Stats	+/-
Absorptive capacity	AC	Human capital and Research & Development Stock.	R&D Stock: Perpetual inventory method to estimate R&D capital stocks from R&D expenditure flows from WDI Human capital index is taken from Penn World Tables 8.0.	+
Protection of property rights	PoPR	Protection of property rights index	Fraser Institute, Economic Freedom	+/-
Quality of Regulations	QoR	Regulations Index	Fraser Institute, Economic Freedom	+/-

Source: Authors.

The descriptive statistics of the variables are presented in Table 3. On average, there is one patent per 10,000 inhabitants in the sample countries. Japan has the highest ratio of patents per capita where there is one patent per 333 inhabitants. The table also shows that on average, there is 0.6 USD of FDI stock per one USD of GDP in the sample countries. Malta has the highest stock of FDI in the sample countries with 19 USD worth of FDI per one USD of GDP. Looking at the protection of property rights and quality of regulations, both variables have mean values greater than the mid-point of 5 of the total scale showing moderate levels of property rights and quality of regulation in the sample countries with low variation. Research and Development stock is on average 18 USD per one USD of GDP however with quite a high variance. The gap between minimum and maximum values of R&D capital as a percentage of GDP is about 70 USD with a standard deviation of 14.5. Keeping high variance of some of the variables used in this analysis, all variables were used in their natural log forms.

**Table 3:** Descriptive statistics.

**Variable**	**Obs**	**Mean**	**Std. Dev.**	**Min**	**Max**
Patents per capita	1,063	0.0005	0.0007	0.0000	0.0042
FDI Stock/GDP	1,063	0.000001	0.000002	0.000000	0.000020
PoPR	1,063	6.674	1.635	3.013	9.339
Quality of Regulation	1,063	6.994	1.145	2.410	9.327
HC	1,063	3.046	0.519	1.450	4.746
R&D Stock/GDP	1,063	20.558	17.485	0.278	89.061

Source: Authors.

## 5. Results and discussion

**Fig 1:**
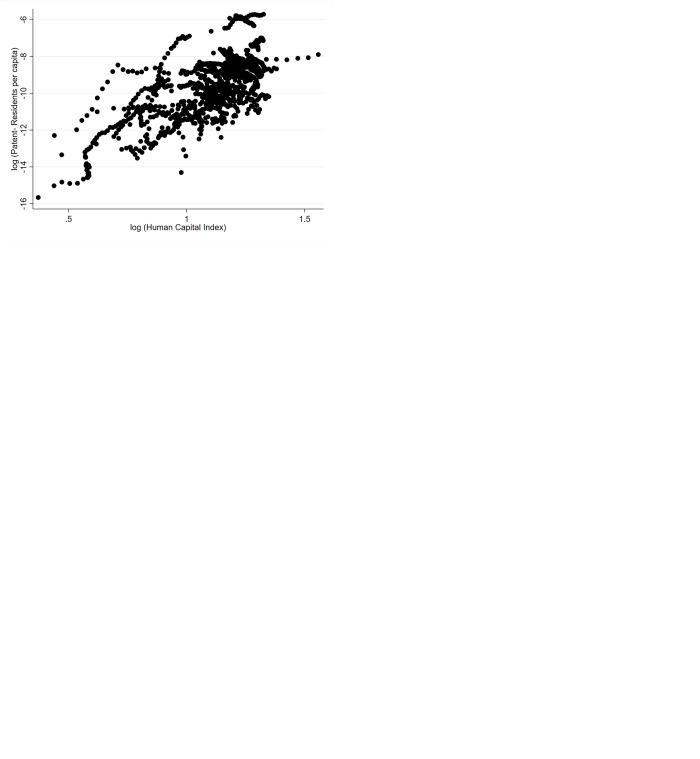
Bi-variate relationships of explanatory variables with the domestic innovation. Source: authors.

Following the expected relationship between each explanatory variable and domestic innovation guided by theory in Table 2 and Fig 1 shows bi-variate relationships using the data for 49 countries and 16 years. All scatter plots show a positive correlation between each explanatory variable and the dependent variable. In particular, R&D Capital stock (a proxy for absorptive capacity) shows a positive correlation with domestic innovation. Similarly, bi-variate scatter plots figure of each explanatory variable including FDI stock, Protection of Property rights, Quality of Regulations and Human capital presents the direct correlation with the predictor variable domestic innovation.

In the following paragraphs, we present and discuss the results of the empirical estimations of Eq 3 and its extensions in Table 4. Column 1 shows the results of the main model using GMM estimation technique. The magnitude of the autoregressive term is less than 1 and it is highly significant, a condition that is necessary for the validity of GMM estimates. Strong property rights protection has a positive and significant relationship with domestic innovation, and so does the quality of regulations. These results are also in line with the expectations based on economic theory (for example Fu [61]. FDI stock is negatively correlated with domestic innovation which supports the findings of Brambilla et al. [17] and Girma et al. [16]. This is consistent with the Schumpeterian effect highlighted by Aghion et al., [18] in which, competition decreases monopoly rents and reduces the incentive to innovate, especially at high levels of competition. It is also possible that in the presence of weak property rights protection, domestic firms prefer to imitate instead of innovating. R&D Capital stock and human capital, the two proxies for absorptive capacity, show a positive and significant relationship with domestic innovation. It shows that, in addition to R&D capital stock being the direct input in the innovation activity, human capital also plays an important role in absorbing external knowledge and generating patents. These findings are consistent with the arguments of Cohen and Levinthal [21,49]. The financial crisis dummy is negative and statistically significant in all models. It shows that the financial crisis harmed innovation activities in the sample countries.

**Table 4:** Estimation results—dependent variables: Patents per capita.

	(1)	(2)	(3)	(4)	(5)
log(Patents per capita)_t-1_	0.906***	0.904***	0.928***	1.010***	0.914***
	(0.0210)	(0.0238)	(0.0199)	(0.0110)	(0.0183)
log(PoPR)	0.530***	0.479**	0.189***	0.340***	0.334
	(0.0985)	(0.211)	(0.0526)	(0.0491)	(0.619)
log(Quality of Regulation)	0.242***	0.280**	2.825**	-0.744***	0.442***
	(0.0534)	(0.135)	(1.366)	(0.105)	(0.119)
log(FDI Stock/GDP)	0.044***	0.0231*	0.377**	0.132**	0.119
	(0.00535)	(0.0135)	(0.185)	(0.0509)	(0.0746)
log(R&D Stock/GDP)	0.100***	-0.00667	0.0267	0.045***	0.00347
	(0.0199)	(0.0511)	(0.0198)	(0.00600)	(0.0160)
log(HC)	0.890***	0.857***	0.392**	1.722**	0.227*
	(0.200)	(0.233)	(0.179)	(0.741)	(0.128)
Financial Crisis Dummy	-0.00968*	-0.009**	-0.0131**	-0.068***	-0.029***
	(0.00556)	(0.003)	(0.00631)	(0.014)	(0.0057)
log(FDI Stock/GDP)* log(R&D Stock/GDP)		0.004**(0.001)			
log(FDI Stock/GDP)*log(Quality of Regulation)			0.204**(0.093)		
log(FDI Stock/GDP)* log(HC)				0.121**(0.049)	
log(FDI Stock/GDP)*log(PoPR)					0.019**(0.006)
	-0.675	-0.101	4.541*	-0.813	-3.801***
Constant	(0.426)	(0.797)	(2.638)	(0.804)	(1.197)
AR(2) p-value	0.493	0.491	0.507	0.479	0.492
Hansen p-value	0.691	0.726	0.701	0.700	0.669
Number of Instruments	110	130	122	72	101
Number of Countries	49	49	49	49	49
Number of Observations	955	955	955	955	955

Standard errors in parentheses

* p<0.10

** p<0.05

*** p<0.01. Insignificant Hansen test statistics show that the instruments are valid and insignificant AR(2) stat shows that there is no second order autocorrelation in the model. The instruments matrix was collapsed and a total of 70 instruments were used.

Source: Authors.

Columns 2 to 5 test our hypotheses 1, 2, and 3. Columns 2 and 4 show the moderating effects of two proxies of AC on domestic innovation. In Column 2, the interaction between R&D capital stock and FDI has a positive sign which shows that where R&D capital stock is higher, the effect of FDI on domestic innovation is stronger. This result shows support for Hypothesis 1 where we expected AC to positively moderate the effect of FDI on domestic innovation. In contrast, Column 4 presents the interaction between human capital (a second proxy of AC) and FDI. The coefficient of this interaction is positive but insignificant. This could be so because the human capital index captures the levels of education in the country which does not necessarily reflect the technological capabilities of the country. Hence, a country would need more than simple average years of schooling to absorb technological knowledge from FDI which is why we found a positive and statistically significant coefficient of the interaction between FDI and R&D capital stock. Column 3 presents the interaction between FDI and regulatory quality which shows that FDI has a positive effect on domestic innovation in countries where regulatory quality is high. Similarly, in Column 5, the interaction between FDI and PoPR shows that FDI positively affects domestic innovation in countries where property rights protection is strong.

The results presented above are quite revealing. The negative relationship between FDI stock and domestic innovation implies that domestic firms move from innovation to imitation and reallocate their resources to the absorption of knowledge instead of innovating themselves. Moreover, a positive and significant coefficient of the interaction between R&D capital stock and FDI shows that the benefits of knowledge spillovers are enjoyed mainly by the firms that invest in R&D. Results also show that level of education does not matter for the absorption of knowledge i.e. knowledge stock has to complement the knowledge that should be absorbed which is why we found the positive and significant moderating effect of R&D in the relationship between FDI and domestic innovation, but not for simple human capital index. The findings also show that the legal and regulatory environment significantly affects the level of innovation in any given country. Where protection of property rights is high, FDI stimulates domestic innovation. Similarly, where the quality of regulation is high, FDI positively affects domestic innovation. Therefore, from the policymaker’s perspective, this study shows that if FDI is encouraged with the aim to increase domestic innovation then domestic R&D investments should also be encouraged to develop the capacity to absorb knowledge.

Column 3 presents the interaction between FDI stock and Quality of Regulation. The positive and significant coefficient of the interaction shows that, with a better quality of regulation, competition created by FDI has an Escape-Competition effect in the domestic market where domestic firms seek incremental profits through innovation [20]. Similarly, a positive and significant coefficient of the interaction between FDI stock and protection of property rights shows that with incoming FDI, domestic innovation output increases. It could be because strong property rights discourage imitation for a set time and domestic firms must innovate to stay competitive in the market. These findings are against the conjecture of Boldrin and Levine [70].

## 6. Conclusion

In this paper, we reexamine the relationship between FDI and innovation. We contribute to the literature on this topic by examining the role of AC, the quality of regulations, and PoPR in the relationship between FDI stock and innovation. We found that FDI generally discourages domestic innovation, perhaps because of the Schumpeterian effect where, due to the decline in monopoly rents, innovation is discouraged. We also found out that countries with strong PoPR, higher absorptive capacity, and good quality of regulations tend to have a higher number of patents. In terms of moderating effects, we found that AC significantly and positively moderates the relationship between FDI and innovation showing that domestic knowledge stock is crucial for the absorption of knowledge through external sources and it helps the domestic economy to transform this knowledge into innovation. However, when AC is proxied by human capital, then there is no moderating effect of AC on the relationship between FDI stock and domestic innovation.

Our results show that R&D should be emphasized to foster innovation in a country. Also, the FDI-domestic innovation relationship becomes positive where countries have strong quality regulations and strong property rights protection. Therefore, the countries, particularly developing, should focus on institutional reforms to facilitate MNEs, and strong protection of property rights to stimulate domestic innovation.

This study has the following limitations. Firstly, patent data is not a perfect measure of innovation, particularly for developing countries. For instance, there is a possibility that innovative firms may not file a patent application to keep technology secrets with innovators in developing countries. Secondly, this study is based on macro-data with a high level of aggregation which overshadows the underlying factors that encourage or discourage innovation. Finally, it was not possible to distinguish between innovative and non-innovative FDI in the data due to which, some of the parameters could be underestimated.
