# Correction: Foreign direct investment and domestic innovation: Roles of absorptive capacity, quality of regulations and property rights

**DOI:** 10.1371/journal.pone.0333528

**Published:** 2025-09-30

**Authors:** The PLOS ONE Staff

An additional affiliation is missing for the second author. Muhammad Ali is also affiliated with the Department of Economics, School of Social Sciences and Humanities, National University of Sciences and Technology, Islamabad, Pakistan.

The Funding statement for this article is incorrect. The correct Funding statement is as follows: This work was funded by the Alexander von Humboldt Fellowship, awarded to Muhammad Ali.

The publisher apologizes for the errors.
